# Study rationale and design of the EANITIATE study (EmpAgliflozin compared to NPH Insulin for sTeroId diAbeTEs) - a randomized, controlled, multicenter trial of safety and efficacy of treatment with empagliflozin compared with NPH-insulin in patients with newly onset diabetes following initiation of glucocorticoid treatment

**DOI:** 10.1186/s12902-020-00561-0

**Published:** 2020-06-15

**Authors:** Carina Kirstine Klarskov, Helga Holm Schultz, Frederik Persson, Tomas Møller Christensen, Thomas Peter Almdal, Ole Snorgaard, Katrine Bagge Hansen, Ulrik Pedersen-Bjergaard, Peter Lommer Kristensen

**Affiliations:** 1 Department of Endocrinology and Nephrology, Nordsjaellands Hospital, Dyrehavevej 29, DK-3400 Hilleroed, Denmark; 2 Department of Oncology at Herlev-Gentofte University Hospital, Herlev Ringvej 75, DK-2730 Herlev, Denmark; 3 Steno Diabetes Center Copenhagen, Niels Steensens vej 2, DK-2820 Gentofte, Denmark; 4 Department of Endocrinology, Bispebjerg University Hospital, Bispebjergbakke 23, DK-2400 Copenhagen, Denmark; 5 Department of Endocrinology, Copenhagen University Hospital (Rigshospitalet), Blegdamsvej 9, DK-2100 Copenhagen, Denmark; 6 Hvidovre University Hospital, Kettegård Alle 30, DK-2650 Hvidovre, Denmark; 7 Faculty of Health and Medical Sciences, University of Copenhagen, Blegdamsvej 3B, DK-2200 Copenhagen, Denmark

**Keywords:** Glucocorticoids, Diabetes, SGLT2-i, NPH-insulin, CGM, RCT

## Abstract

**Background:**

A well-known metabolic side effect from treatment with glucocorticoids is glucocorticoid-induced diabetes mellitus (GIDM). Guidelines on the management of GIDM in hospitalized patients (in the non-critical care setting), recommend initiation of insulin therapy. The scientific basis and evidence for superiority of insulin therapy over other glucose lowering therapies is however poor and associated with episodes of both hypo- and hyperglycaemia. There is an unmet need for an easier, safe and convenient therapy for glucocorticoid-induced diabetes.

**Methods:**

EANITIATE is a Danish, open, prospective, multicenter, randomized (1:1), parallel group study in patients with new-onset diabetes following treatment with glucocorticoids (> 20 mg equivalent prednisolone dose/day) with blinded endpoint evaluation (PROBE design). Included patients are randomized to either a Sodium-Glucose-Cotransporter 2 (SGLT2) inhibitor or neutral protamin Hagedorn (NPH) insulin and followed for 30 days. Blinded continuous glucose monitoring (CGM) will provide data for the primary endpoint (mean daily blood glucose) and on glucose fluctuations in the two treatment arms. Secondary endpoints are patient related outcomes, hypoglycaemia, means and measures of variation for all values and for time specific glucose values. This is a non-inferiority study with the intent to demonstrate that treatment with empagliflozin is not inferior to treatment with NPH insulin when it comes to glycemic control and side effects.

**Discussion:**

This novel approach to management of glucocorticoid-induced hyperglycemia has not been tested before and if SGLT2 inhibition with empaglifozin compared to NPH-insulin is a safe, effective and resource sparing treatment for GIDM, it has the potential to improve the situation for affected patients and have health economic benefits.

**Trial registration:**

www.clinicaltrialsregister.eu no.: 2018–002640-82. Prospectively registered November 20th. 2018.

**Date of first patient enrolled:** June 4th. 2019.

This protocol article is based on the EANITATE protocol version 1.3, dated 29. January 2018.

## Background

Glucocorticoid treatment is frequent in inpatients and outpatients in medical specialties as rheumatology, oncology, haematology, neurology, nephrology, and pulmonology. A well-known metabolic side effect from treatment with glucocorticoid is hyperglycaemia and diabetes (and worsening of pre-existing diabetes). The acute symptoms of diabetes include general discomfort, polyuria, thirst, and blurry vision. Moreover, high plasma glucose during hospitalisation is associated with increased risk of inflammation, infection [1] and mortality [2, 3] and glucocorticoid induced diabetes (GIDM) is associated with increased mortality [4].

The diabetic effect of glucocorticoids relies partly on induction of insulin resistance in the liver, skeletal muscles and adipose tissue. In addition, glucocorticoids may reduce beta-cell function, reduce the insulinotropic effects of incretin hormones and increase glucagon levels. In concert, these changes lead to increased plasma glucose concentration [5].

The prevalence of GIDM depends on the population studied and the duration and dose of glucocorticoid. In a review from 2018 including 13 very heterogeneous studies, the overall incidence of GIDM was 18.6% [6]. The glucocorticoids were administered in different regimens, on different indications, and for both malignant and non-malignant diseases.

Guidelines on detection and management of hyperglycaemia in hospitalized patients (in the non-critical care setting) recommend initiation of sliding scale insulin (aspart) therapy or other insulin regimens in case of hyperglycaemia during glucocorticoid treatment [7, 8]. The evidence for the superiority of this treatment is however poor and it is a clinical experience that insulin treatment during admission to hospital is associated with episodes of both hypo- and hyperglycaemia. Insulin is considered a high-risk treatment, classified from the Danish authorities’ stratification [9].

Initiation of insulin treatment in patients without previously known diabetes is associated with a need for training in blood glucose measurements and insulin injection. Moreover, at discharge from hospital to home, healthcare providers must educate the patient, the family and potential caregivers in different aspects of diabetes self-management, which is an unwelcome burden on top of existing stress and anxiety in relation to the background disease requiring glucocorticoid treatment. Focus on adjustment of insulin doses is also required if the glucocorticoid dose is changed or the therapy is stopped and demands multiple contacts between patient/family and hospital or health care providers in primary care.

Hence, there is an unmet need for an easier, safe and convenient therapy for GIDM. Oral blood glucose lowering treatment of GIDM with empagliflozin - an SGLT2 inhibitor - once daily is a new approach. Empagliflozin lowers plasma glucose and the risk profile is beneficial. SGLT2 inhibitors inhibit the SGLT2 receptor in the proximal tubule in the kidney, preventing the reabsorption of glucose which leads to glucosuria. As glucose is excreted in urine, plasma levels decrease leading to an improvement in glycaemic parameters [10]. The risk of hypoglycaemia is very low with these agents (since the glucose lowering effect of the drugs is low at normoglycaemia), hence frequent glucose measurements are not needed. Moreover, there is no need for dose changes. Altogether, treatment with empagliflozin may - compared to insulin treatment - reduce risk of hypoglycaemia and work load in relation to glucose measurements and insulin dose changes. Moreover, patients’ and relatives’ workload and diabetes-related anxiety may be reduced since the treatment with empagliflozin is simpler.

## Methods/design

### Aims

Our study is designed to test the safety and efficacy of empagliflozin for the treatment of GIDM compared to neutral protamine Hagedorn (NPH) insulin. The hypothesis is that empagliflozin can be used as a safe alternative to NPH insulin in patients with GIDM. The primary aim is to compare mean glucose level (primary endpoint, measured by continuous glucose monitoring (CGM)) between empagliflozin and NPH insulin treatment groups, with a tolerated significant difference in mean daily glucose of up to 2 mmol/L to the higher side.

Secondary endpoints are 9 other CGM metrics, based on recommendations endorsed internationally (American Diabetes Association, American Association of Clinical Endocrinologists, American Association of Diabetes Educators, European Association for the Study of Diabetes, Foundation of European Nurses in Diabetes, International Society for Pediatric and Adolescent Diabetes, JDRF, and Pediatric Endocrine Society) [11], Self-Monitoring of Blood Glucose (SMBG) -related outcomes, and patient-related outcomes.

Tertiary outcome is cost-effectiveness of empagliflozin compared to NPH-insulin in treating GIDM.

Further predefined study aims are exploratory and include differences between treatment groups in relation to HbA_1c_, fructosamine, performance status [12].

### Study design

**Fig. 1:**
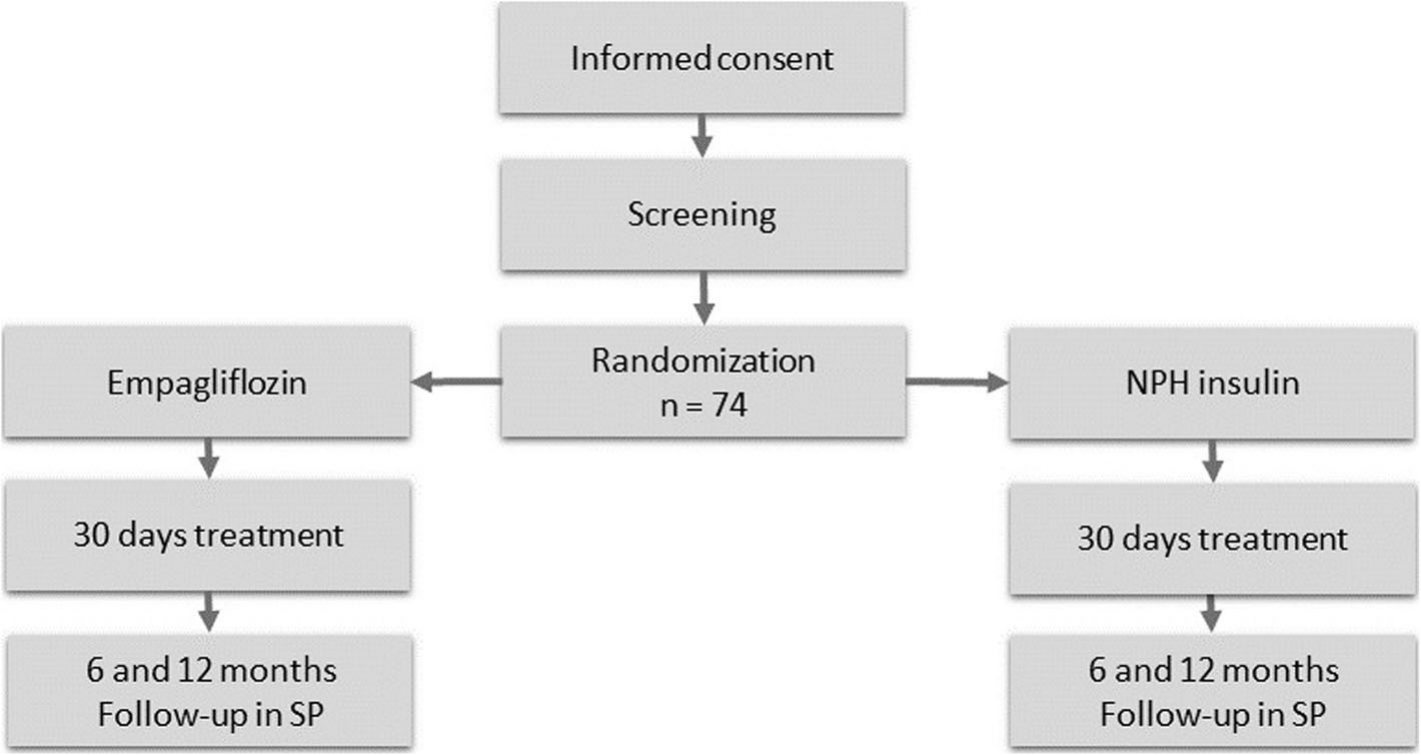
EANITIATE study design flow chart. SP = Sundhedsplatformen (Electronic patient records). A complete list of study sites can be obtained from the corresponding author

Open, prospective, multicenter, GCP-monitored, randomized (1:1), parallel group study, in patients with new-onset diabetes following recently initiated treatment with glucocorticoids (> 20 mg equivalent prednisolone dose/day) with blinded endpoint evaluation (PROBE design). Patients in need of treatment of glucocorticoid-induced diabetes mellitus are invited and randomized one of two different treatments: NPH insulin (Insulatard®) or empagliflozin for 30 days, see Fig. 1. Pre-specified rules on rescue medication with sliding scale insulin (aspart) for patients in both treatment arms are defined. Blinded CGM will provide data for the primary and secondary endpoints.

This is an open study since it is difficult to mask tablets vs. injections. Randomization (1:1) will minimise bias and participants will be stratified by dose of glucocorticoids (20 mg - 75 mg and > 75 mg). To further minimize bias, CGM-monitors and endpoint evaluations will be blinded.

If glucocorticoid treatment is discontinued, glucose levels will still be checked by self-monitored blood glucose 4 times a day and if in the normal range for 2 days (equivalent to 8 measurements) the study treatment will be discontinued. Glucose levels will be closely monitored 4 times a day for the rest of the 30-day trial period, and if hyperglycemia evolves, the patient will be treated with the investigational product (IP) again. After glucocorticoid treatment is stopped, a project nurse will be in touch with the patient until the glucose levels are in the normal range for 3 days in a row, or until the 30 days trial period is over. A plan for any further treatment or monitoring after the 30-day trial period will be planned at the last visit of the patient.

If a patient discontinues the IP, the patient will be replaced by further recruitment to maintain the required total minimum of 50 evaluable patients. Discontinuation of IP due to normoglycaemia after treatment with glucocorticoids is stopped within the 30-day treatment period will not be withdrawals and the visits will continue as planned.

### Rationale for study design and endpoints

GIDM is different from other types of diabetes because the primary feature is postprandial hyperglycaemia and fasting blood glucose can often be normal. Often the hyperglycaemia starts within the first 1–3 days of glucocorticoid treatment – thus with a 30-day trial period, HbA_1c_ will be unreliable as an endpoint, as HbA_1c_ reflects the average blood glucose during the last 8–12 weeks. Fasting glucose level will also be unreliable, as glucocorticoids are mostly given in the morning and thus the effect on plasma glucose usually begin before dinner [13] and diminish during night. Thus, the primary outcome measure is the difference between treatment arms in mean glucose from CGM in the first 14 days of treatment. Patients will wear a blinded CGM system on day 0–14. CGM systems provide subcutaneously glucose values every 5 min, which via algorithms is “translated” into plasma glucose values. These data provide us with a unique possibility to gain knowledge about the glucose fluctuations during day and night in patients with GIDM in the two treatment arms. No previous trials have gathered blinded CGM data on patients with GIDM. In addition, the CGM data can form the basis of a mathematical model which make it possible - at least to some extent - to predict glucose values throughout the day and night in this patient population. This could be helpful for patients and healthcare professionals when planning treatment and blood glucose measurements.

### Treatment arms

1. Empagliflozin 10 mg, one tablet daily in the morning.

Rationale for dose selection: All patients in the empagliflozin arm will take 10 mg per day. Empagliflozin is also available in 25 mg. The difference in HbA_1c_ (%) is a reduction of − 0.11 for 25 mg vs 10 mg empagliflozin. The risk of urogenital infections (the most common side effect) is not increased using the high dose of empagliflozin [14, 15].

If the patient tolerates 10 mg per day well and needs additional glucose control, the dose will be increased to 25 mg per day. It is up to the local investigator to make this judgement on an individualized basis.

2) NPH insulin s.c.

Individual need is normally between 0,3–1,0 IE/kg/day [16]. All patients will start with 0,3 IE/kg/day before breakfast. If morning fasting blood glucose is > 10 mmol/L, dose will be increased with 4 IE pr. mmol/L above this limit. Change in dose will not be considered before day 3 unless blood glucose is above 15 mmol/L. If dose exceeds 30 IE pr. day, dosage will be split into 2 with 2/3 before breakfast and 1/3 before dinner [17].

In case of persisting uncontrollable diabetes (defined as plasma glucose values over 20 mmol/L on two consecutive days), the participant should initiate supplementing sliding scale insulin (aspart) treatment up to 5 times per day.

### Discontinuation of IPs

Reasons to discontinue:

- Wrongful randomization.
- Patient decision.
- AE, SAE etc. that contradicts further IP such as diabetic ketoacidosis.
- Non-compliance as judged by the investigator.
- Pregnancy.
- Hypoglycaemia defined as plasma glucose < 4 mmol/L at two different times in 24 h with at least 1 h between.
- Severe hypoglycaemia (need for assistance from others to restore blood glucose).
- Individual risk assessment of volume depletion/hypotension in acute illness, as judged by the investigator.
- Normalization of blood glucose after GK treatment is terminated.

### Discontinuation of GKs

If GK treatment is discontinued, glucose levels will still be checked by SMBG 4 times a day and if in the normal range for 2 days (equivalent to 8 measurements) the IP will be discontinued. Glucose levels will be closely monitored 4 times a day for the rest of the 30-day trial period, and if hyperglycemia evolves, the patient will be treated with the IP again. After GK treatment is stopped, a project nurse will be in touch with the patient until the glucose levels are in the normal range for 3 days in a row, or until the 30 days trial period is over. A plan for any further treatment or monitoring after the 30-day trial period will be planned at the last visit of the patient.

### Withdrawal from study

If a patient discontinues the IP, the patient will be replaced by further recruitment to maintain the required total minimum of 50 evaluable patients. Discontinuation of IP due to normoglycaemia after treatment with glucocorticoids are stopped within the 30-day treatment period will not be withdrawals and the visits will continue as planned.

### Rationale for treatments

The rationale for choosing SGLT2 inhibition as treatment modality in this study has been the convenience of a once daily tablet that is considered a low risk treatment. The side effects are primarily risk of urogenital infections, which will be registered as an adverse event of interest. The risk of hypoglycaemia is very low, as empagliflozin works in an insulin-independent way, by inhibiting the selective reuptake of glucose in the kidney. There are no interactions with other medications that need to be avoided and thus there is no need for change in concomitant medications - but close monitoring with antihypertensive drugs, diuretics and other glucose lowering medications is advised. Empagliflozin also has other beneficial effects independent of the glucose lowering effect - these include reduction of cardiovascular death and improvement of renal outcomes in patients with known diabetes and cardiovascular disease [15, 18, 19]. The rationale for comparing empagliflozin to NPH insulin is that it is a well-known basal insulin regimen that - like empagliflozin - can be given once daily in the morning. Furthermore, NPH insulin was chosen due to the pharmacodynamic profile that in theory - and also in clinical practice - offers the best treatment of glucocorticoid induced hyperglycemia which – when glucocorticoids are prescribed once-daily in the morning - is present during the day and to a much lesser degree during late evening and night [13]. Long-acting insulin analogues are superior to NPH in patients with type 1 diabetes, however this does not appear to be true for steroid induced diabetes [20]. NPH insulin is recommended in numerous publications to control glucocorticoid induced diabetes [21, 22]. The safety concern is primarily hypoglycaemia.

### Patient selection

The target population is hospitalized patients, or equal to being hospitalized as judged by the local investigator, with newly onset GIDM, defined by a fasting plasma glucose of above 7 mmol/L or a plasma glucose of 11.1 mmol/L or more at two different times or one increased glucose value AND hyperglycaemic symptoms after receiving glucocorticoid in a dose equivalent to > 20 mg prednisolone. The participant must not have had any type of diabetes before, or any pancreatic diseases. It is permitted that the participant receive treatment with p.n. insulin prior to the screening procedure (for up to a week), if it is estimated that the effect has worn off – e.g. if a patient has received a dose of p.n. insulin the night before the screening procedure is done.

Potential participants will be recruited by trained diabetes nurses, which are part of diabetes service teams in the participating hospitals, that are already in contact with patients with new-onset GIDM. Patients will be asked if they are interested in information about the trial, and if so, they will be informed in accordance with the Danish Regional Committee on Biomedical Research and Ethics. If the patient agrees to participate and signs the required forms, the project nurse will be contacted, and he or she will then perform the screening procedure, and if enrolled also the randomization in REDcap® (a web-based electronic CRF).

The study population is expected to suffer from a wide range of underlying diseases as patients will be screened from multiple centres and departments. The participants must have an eGFR of 60 ml/min/1.73m^2^ or above as the glucose-lowering effect of an SGLT2 inhibitor has been shown to be impaired with lower eGFRs [23]. If the investigator estimates, that the patient is in a state where the most recent eGFR is unreliable (acute kidney injury, dehydration, other), the patient cannot be included.

### Inclusion and exclusion criteria

Inclusion criteria: Written signed informed consent prior to any study specific procedures, recent (within a week) diagnosis of GIDM (defined as non-fasting plasma glucose measured > 11.1 mmol/L at two different occasions, OR at one occasion above 11.1 mmol/L with classical hyperglycaemia symptoms, OR a fasting plasma glucose of > 7 mmol/L), hospitalized or equal to being hospitalized as judged by the local investigator at the time of screening, patients > 18 and < 85 years at the time of consent, eGFR ≥60 ml/min/1.73m^2^ (estimated by CKD-EPI formula) at visit 1, female patients in childbearing age must use appropriate contraception as described by the Danish Medicines Agency, must be able to communicate with the study personnel.

Exclusion criteria: Known diabetes (treated or not treated) prior to initiation of glucocorticoid treatment, use of any blood glucose-lowering medication for the last 30 days prior to the trial for any reason, except receiving insulin prior to screening for up to 1 week, hyperglycaemia with a glucose level > 20 mmol/L, any former or ongoing pancreatic disorder, known or suspected hypersensitivity to trial product(s) or products with the same content/known cross-reactivity, females who are pregnant, breast-feeding, intend to become pregnant or are not using adequate contraceptive methods, the receipt of any investigational product 30 days prior to this trial, known or suspected abuse of alcohol or drugs, suspected non-compliance with the protocol (as judged by the investigator), involvement in the planning/or conduct of the study, previous randomization in the study, participation in another study with an investigational product during the last 30 days prior to enrolment.

### Data collection and randomization procedure

A web based electronic CRF (REDcap®) is provided and all data related to the trial will be recorded in here and provide the basis for a central database. Randomization (1:1) in REDcap® is stratified by dose of glucocorticoid at the day of the screening visit in blocks of 2,4 and 6. The patient will belong to one of two groups: Group 1: Glucocorticoid dose equivalent to 20–75 mg prednisolone per day. Group 2: Glucocorticoid dose equivalent to more than 75 mg prednisolone per day. No further stratification will be done to ensure a ‘real life’ study.

Randomization in REDcap® was set up by independent personnel not involved in other parts of the trial.

### Visit procedures

**Fig. 2:**
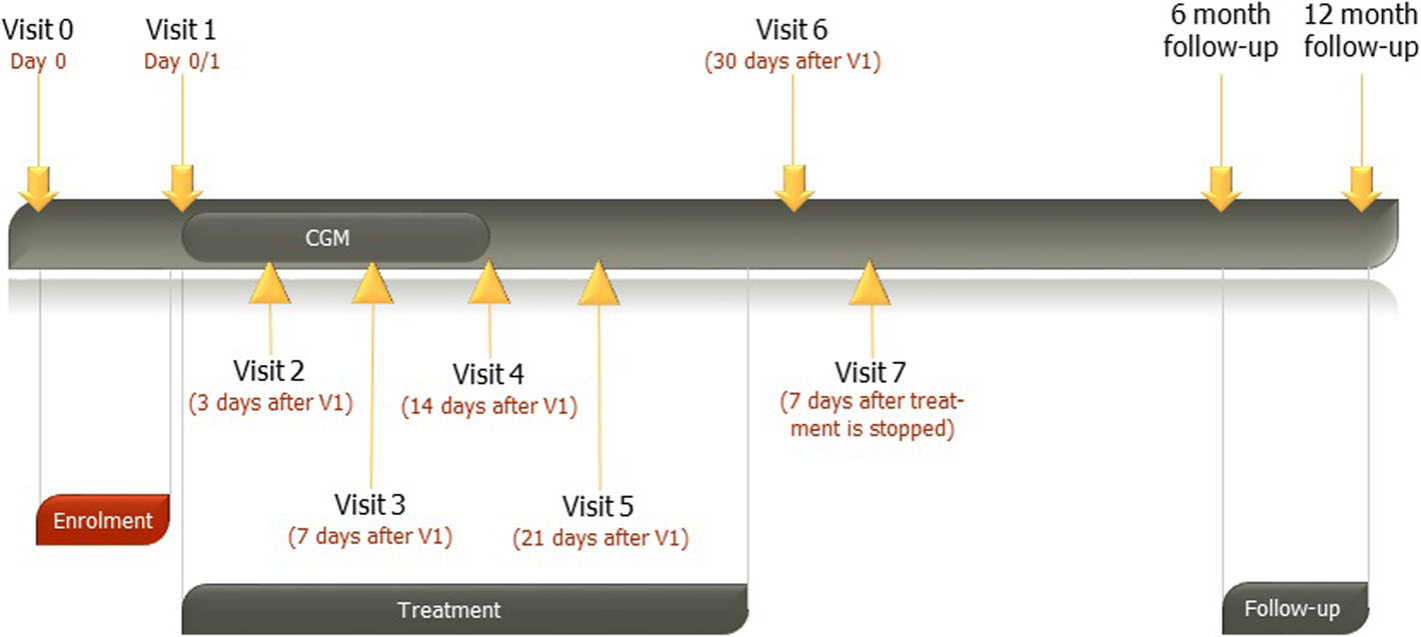
EANITIATE visit timeline

Patients are seen in the hospital setting at 5 University Hospitals in Copenhagen, Denmark for visit 0–7 see Fig. 2 and Table 1. If not hospitalized, patients will be seen at the out-patient clinics at the abovementioned locations. Prior to any protocol-related procedures written informed consent is obtained. The patient is screened for in- and exclusion criteria at visit 0 and information about demography, medical and surgical history, family history of type 1 and 2 diabetes mellitus, current medication, indication for glucocorticoid treatment and dose of glucocorticoid, date of diagnosis of GIDM, vital signs: weight, height, blood pressure, pulse, general physical examination, volume status (lung auscultation and peripheral oedema examination (pitting) and weight), laboratory tests: Haemoglobin, fructosamine, HbA_1c_, plasma glucose, C-peptide, leukocytes with differential count, CRP, platelet count, iron, transferrin, MCV, reticulocytes, albumin, ALAT, ASAT, alkaline phosphatase, LDH, bilirubin, calcium-ion, lipids, creatinine, potassium, sodium, urea. Finger prick ketones, genetic research material collection for future biobank (plasma, serum, whole blood), pregnancy test in women of child-bearing potential with a standard dip-stick and urine test (signs of urinary tract infection).

**Table 1:**
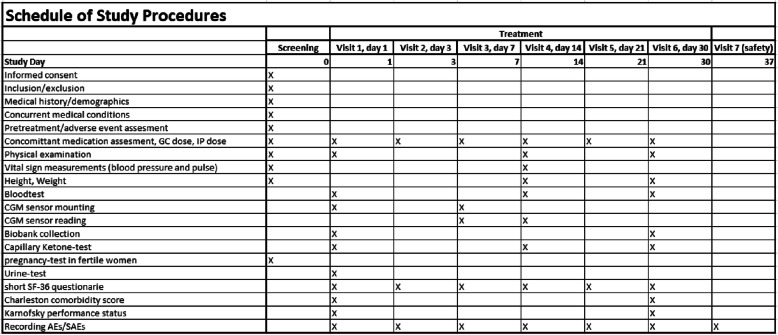
Table 1.

The investigator will prior to visit 1 assess eligibility from the laboratory results (eGFR and fluid balance). If not eligible, patients will not be allowed to continue to visit 1 (screen failures).

Visit 1: general physical examination, vital signs (blood pressure and pulse), concomitant medication, including dose of glucocorticoid, recording of AE’s or SAE’s since last visit if not on same day as enrolment, randomization in REDcap®, dispensing of IP’s. Instructions by project nurse in glucose measurements, study drug use, reporting to research staff, CGM-sensor is attached. Short SF-36 Questionnaire in REDcap® (The short SF-36 Health Survey is a 36-item validated patient-reported survey of patient health), Visual Analogue Rate Scale of Health-related quality of life (Monitoring of patient-reported current health on a scale from 0 to 100), Charlson comorbidity score, Karnofsky Performance status.

Visit 2 (telephone visit - 3 days after visit 1): Instructions by project nurse in glucose measurements and study drug use are repeated, recording of AE’s or SAE’s since last visit, Short SF-36 Questionnaire in REDcap®, VAS, glucocorticoid dose, study drug dose.

Visit 3 (7 days after visit 1): Removal of CGM, attachment of new CGM, recording of AE’s or SAE’s since last visit, Short SF-36 Questionnaire in REDcap®, VAS, glucocorticoid dose, study drug dose.

Visit 4 (14 days after visit 1): Removal of CGM. Gathering data about: Glucose measurements, symptoms of hyperglycaemia and side-effects of medication, episodes of severe hypoglycaemia, number of contacts between patient and general practitioner due to diabetes related problems, other hospital physicians and any admissions to hospital. General physical examination and vital signs, laboratory tests, recording of AE’s or SAE’s since last visit, Short SF-36 Questionnaire in REDcap®, VAS, glucocorticoid dose, study drug dose.

Visit 5 (Telephone visit - 21 days after visit 1): As visit 2.

Visit 6 (30 days after visit 1): Recording of AE’s or SAE’s since last visit, glucocorticoid dose, study drug dose. Laboratory tests are repeated, finger prick ketones, future biobank sample collection (plasma, serum, whole blood), vital signs, Short SF-36 Questionnaire in REDcap®, VAS-scale, Charlson comorbidity score, Karnofsky Performance status.

Visit 7 (safety follow-up - 7 days after trial medication is stopped).

Recording of AE’s or SAE’s since last visit, evaluation of prior AE’s or SAE’s and follow-up on any safety concerns.

When a patient is no longer in the trial, their glycaemic target is based on local guidelines which is currently 5-10 mmol/L in all participating centers.

Follow-up.

Without further obligatory visits, patient status will be assessed at 6 and 12 months via the electronic patient record Sundhedsplatformen (SP), which covers all patients and hospitals in the Capital Region of Copenhagen (Region Hovedstaden) and the region of Sjælland (Region Sjælland). Number of admissions is counted and cause of admission (hyperosmolar hyperglycaemic state (HHS), infections, acute kidney injury, bacteraemia, and other) is assessed. Also, status on death is assessed.

### Early termination of the trial

The Sponsor reserves the right to terminate the trial under the following conditions:

1. Safety concerns.
2. Proven lack of efficacy.

If the trial is prematurely terminated or suspended, the investigator should promptly inform the subjects and ensure appropriate therapy and follow-up. Furthermore, the investigator and/or sponsor should promptly inform the pertinent ethics committee and regulatory authorities.

### Adverse events reporting

Information about Adverse Events (AEs), whether reported by the subject, discovered by the investigator by reviewing diary records, detected through physical examination, laboratory test or other means, must be collected and recorded on the AE form and followed up as appropriate.

Evaluation of AEs including severity, causality, outcome and seriousness assessments must be performed by a physician. Any AE occurring from the time the informed consent was signed by the subject and until 7 days after the last treatment day must be recorded and reported on an AE page in the eCRF*.* Standardised report forms for AEs and SAEs is provided as part of the eCRF.

### Definition of endpoints

#### Primary objective

To determine if empagliflozin can be used as a safe alternative to NPH insulin in patients with GIDM, with a tolerated significant difference in mean daily glucose (mean of CGM measurements) of up to 2 mmol/L to the higher side. Outcome measure: Mean glucose difference between the empagliflozin and NPH insulin group (calculated mean of CGM glucose profiles over the 2 first weeks of treatment). Calibration capillary glucoses values will be recorded. 70% of possible data (10 out of 14 days or 2822 out of 4032 possible tests) should be available for the patient to be included in the statistical analysis [11].

#### Secondary objectives

To determine if empagliflozin is non-inferior (in the below outcome categories) compared with NPH insulin in treating GIDM for the following CGM based outcome measures:

##### TIR metrics

Between group differences in Time spent In Range (TIR) 3.9–10 mmol/L.

Between group differences of time glucose is above range (TAR) 10–13.9 mmol/L and 13.9–22.2 mmol/L.

Between group differences of time glucose is below range (TBR) 3–3.9 mmol/L and < 3 mmol/L.

##### Glucose exposure metrics

Between group differences in AUC for blood glucose during periods when blood glucose levels reach 10–13.9 mmol/L and 13.9–22.2 mmol/L.

Between group differences in AUC for blood glucose during periods when blood glucose levels reach 3–3.9 mmol/L and < 3 mmol/L.

Between group differences in mean daytime blood glucose levels.

Between group differences in mean nocturnal blood glucose levels.

Between group differences in eHBA_1c_.

##### Glycemic variability metrics

Between group differences in SD of 24-h blood glucose values.

Between group differences in the SD of daytime blood glucose values.

Between group differences in SD of nocturnal blood glucose values.

Between group differences of MAGE (mean amplitude of glycemic excursions).

Between group difference in glucose variability measured by the coefficient of variation (CV).

##### Other metrics

Between group differences in number of hypoglycemic events in total and divided into levels (3–3.9 mmol/L, < 3 mmol/L or a need for third party assistance to restore blood glucose).

and nighttime/daytime.

Between group differences in number of hyperglycemic events in total and divided into levels and nighttime/daytime.

Hypo- and hyperglycemic events are defined as at least 15 min spent in the specific range and each event must be at least 30 min apart. Nighttime is defined as midnight – 6. am [11].

#### Other secondary outcomes

1) Number of patients in each group that reach a daily mean glucose level of 6–12 mmol/L during treatment for at least 7 out of the first 14 days of treatment. Outcome measure: CGM and SMBG mean glucose levels. SMBG outcome measure: Mean plasma glucose difference between the empagliflozin and NPH insulin group. Patients will both fill out a diary and the data will also be downloaded directly from the patients glucometers. A minimum of 15 complete 4-point profiles pr. patient will be needed (out of 30) for the patient to be included in this secondary endpoint statistical analysis. The capillary glucoses are to be taken: 1) morning fasting 2) preprandial noon 3) preprandial dinner 4) bedtime.

2) Number of events of mild hypoglycaemia and severe hypoglycaemia (need for third party assistance to restore blood glucose).

3) Quality of life: Outcome measures: PRO from questionnaires including VAS-rating, interviews and information from electronic patient record.

#### Exploratory objectives

To compare the effect of empagliflozin versus NPH insulin on:

1. The Karnofsky performance status score
  Outcome measure: Change from baseline to 14 and 30 days in Karnofsky Performance status score [12].
2. Health-related quality of life from the short SF-36 Health Survey, an 36-item validated patient-reported survey of patient health [24]. Outcome measure: Change from Baseline to 30 days in the short SF-36 questionnaire summary physical score (PCS) and summary mental score (MCS).
3. Changes from Baseline in Visual Analogue Scale (VAS), interindividual and in between the two groups. The VAS has been extensively used in the evaluation of health-related quality of life (HRQL). The VAS is used to monitor patient-reported current health on a scale from 0 to 100 [25].
4. Fructosamine (FA) levels at day 1, 14 and 30. Outcome measure: Differences in FA changes between the 2 groups from laboratory results at day 1, 14 and 30. FA was chosen as it reflects the mean glycemic control in a shorter time (2–3 weeks) compared with glycated hemoglobin (HbA_1C_). HbA_1c_ and FA levels are highly correlated. Because FA reflects glycated S-proteins, results from patients with albumin out of the normal range cannot be included in the analysis [26].
5. Between group differences in HbA_1c_ changes based on their baseline HbA_1c_ to 30 days.
6. Effect on systolic BP. Outcome measure: Change in systolic BP from baseline.
7. Bodyweight. Outcome measure: change in bodyweight from baseline.

#### Tertiary objective

To determine the cost-effectiveness of empagliflozin compared with NPH insulin in treating GIDM. Outcome measure: Health economic analyses will estimate the cost of health care expenses in the two treatment arms. Incremental cost–effectiveness ratio (ICER) analysis will be conducted [27]. ICER analysis compares the incremental costs associated with one intervention (empagliflozin) over another (NPH insulin) with the incremental outcome costs (negative or positive) of one intervention over another. In this study the outcome includes but not limited to costs of: Investigational products, blood glucose monitoring, expenses for hospitalization.

#### Safety objectives

To evaluate the safety and tolerability of empagliflozin compared with NPH insulin in treating GIDM. Outcome measures: SAEs. Discontinuation of investigational product (IP) due to adverse events. Changes in clinical chemistry/hematology parameters including finger prick ketones. AEs of interest (urogenital infections, volume depletion, diabetic ketoacidosis (DKA), HHS, hypoglycemic events and other AEs needing any treatment. Number of contacts between patient and general practitioner due to diabetes-related problems, and any admissions to hospital.

Percentage in each group that needing rescue medication due to hyperglycaemia. Outcome measure: Interviews and SP. Dose of sliding scale needed.

### Statistics

#### Power

A total of 74 patients should be included to allow randomization of approximately 37 patients in each arm, and for at least 29 patients to complete 30 days of treatment per arm, with alpha set to 0.05 and beta 0.90. It is estimated that a total of 58 completing patients can provide 90% power to detect a significant difference (two-tailed p-value ≤0.05) between the two treatments in reduction of plasma glucose, if there is a true difference of 2 mmol/L (non-inferiority limit) with a standard deviation (SD) for plasma glucose of 2.6 mmol/L. In our own recent study, which included 1131 blood glucose measurements in patients with newly diagnosed GIDM, we found an SD of 4.2 mmol/L [13]. In another study with 21 patients that developed GIDM after prednisolone, an SD of 1 mmol/L was calculated [28]. Thus, because the true SD for glucose for GIDM is not known, the SD used for the power calculation was pragmatically calculated as a mean from the 2 studies.

It is expected that up to 16 patients (25%) may leave the study prematurely, thus a total minimum of 74 patients will be included and at least 58 complete the study.

### Statistical analysis plan

Before the randomization code will be added to the cleaned dataset, a thorough plan for statistical analysis will be elaborated and accepted by the statistician and investigators. Intention-to-treat (all randomized subjects) and per-protocol statistical analysis will be conducted by the investigators (responsible: Carina Kirstine Klarskov), in collaboration with a statistician. All data will be described including data-incompleteness as well as reasons for data-incompleteness. Data will be analyzed blinded by the daily coordinator of the study Carina Kirstine Klarskov in collaboration with the principal investigator Peter Lommer Kristensen. Any changes to the statistical analysis plan will be described in any future publications.

#### Data presentation

Numeric data will be shown as mean or medians with inter-quartile ranges (IQR) or ranges where relevant. Frequencies will be shown as numbers with percentages and 95% confidence intervals (CI) where relevant. All statistical tests will be 2-tailed and *p* < .05 considered statistically significant. Any missing data will be presented in the appendix of the main manuscript.

#### Primary analysis

A comparison between groups of differences in mean daily plasma glucose from 14-day CGM using a T-test with a 95% confidence interval. If we, based on the primary analysis, can be 95% sure that the biggest difference in glucose is not more than 2 mmol/L then non-inferiority for the experimental arm (empagliflozin) is concluded.

Subgroup analysis will be done with ANOVA, which will include stratification on dose of glucocorticoids and more.

#### Secondary analysis

Continuous variables analysis will be done using a T-test. For categorical outcomes a Chi-square test will be used.

#### Tertiary analysis

Incremental cost–effectiveness ratio (ICER) analysis will be conducted. The incremental costs associated with empagliflozin and NPH insulin will be compared with the incremental outcomes. Intention to treat (ITT) and per protocol (PP) analysis will both be analysed.

## Discussion

EANITIATE is the first combined in-hospital and outpatient study to compare an SGLT2 inhibitor with NPH insulin to treat GIDM. This novel approach to management of glucocorticoid-induced hyperglycemia has not been tested before and if SGLT2 inhibition with empagliflozin compared to NPH insulin is a safe, effective and resource sparing treatment for GIDM, it has the potential to improve the situation for affected patients. Furthermore, it could have health economic benefits.

## Data Availability

The datasets used and/or analysed during the current study are available from the corresponding author on reasonable request.

## Abbreviations

AE: Adverse events
ALAT: Alanine aminotransaminase
ASAT: Aspartate aminotransferase
AUC: Area under the curve
BP: Blood pressure
CGM: Continuous glucose monitoring
CRF: Case report form
CRP: C-reactive protein
CV: Coefficient of variation
DKA: Diabetic ketoacidosis
EU: European union
GCP: Good clinical practice
GIDM: Glucocorticoid-induced diabetes mellitus
GC: Glucocorticoid
HHS: Hyperosmolar hyperglycaemic state
HRQL: Health-related quality of life
ICF: Informed consent form
ICH-GCP: International conference on harmonisation-good clinical practice
IP: Investigational product
LDH: Lactate dehydrogenase
MAGE: Mean amplitude of glycemic excursions
MCV: Middle cell volume
MCS: Mental component summary
MD: Medical doctor
NPH: Neutral protamine hagedorn
P: Pulse
PCS: Physical component summary
PRO: Patient-related-outcome
PROBE: Prospective randomized open blinded end-point
SAE: Serious adverse events
SD: Standard deviation
SF-36: Short form 36
SP: Sundhedsplatformen (Electronic patient records)
TAR: Time above range
TBR: Time below range
TIR: Time in range
VAS: Visual analogue scale
VEK: The regional committee on biomedical research ethics
